# Family structure and breakfast consumption of 11-15 year old boys and girls in Scotland, 1994-2010: a repeated cross-sectional study

**DOI:** 10.1186/1471-2458-12-228

**Published:** 2012-03-22

**Authors:** Kate A Levin, Joanna Kirby, Candace Currie

**Affiliations:** 1 Child and Adolescent Health Research Unit, School of Medicine, University of St Andrews, North Haugh, St Andrews, KY16 9TF, UK; 2 Ludwig Boltzmann Institute for Health Promotion Research, Vienna, Austria

**Keywords:** Breakfast, Nutrition, Adolescent, Socioeconomic factors, Multilevel modelling, Survey methodology

## Abstract

**Background:**

The benefits of breakfast during childhood and adolescence have been reported previously though few studies have considered family structure inequalities in breakfast consumption. The proportion of young people living in non-traditional family types has increased in recent years, strengthening the need to describe and monitor the impact of the changing family unit on adolescent breakfast consumption. This study aimed to describe changes in daily breakfast consumption among adolescents in Scotland between 1994 and 2010, while also considering family structure inequalities, and the degree to which these have changed over time.

**Methods:**

Data from the 1994, 1998, 2002, 2006 and 2010 Scottish Health Behaviour in School-aged Children (HBSC) surveys were analysed using logistic multilevel regression models for binary outcome variable daily breakfast consumption.

**Results:**

Daily breakfast consumption among adolescents increased between 1994 and 2010, although there were differences by age and sex. In fact those aged over 14.5 years saw decreases in breakfast consumption, and girls saw significantly larger increases than boys. Daily breakfast consumption was more prevalent among adolescents from 'both parent' families, with lowest prevalence among those from single parent families. Trends in daily breakfast consumption between 1994 and 2010 also varied by family structure. While prevalence of daily breakfast consumption increased among those living with 'both parents', the largest proportion of the population, prevalence decreased over time among adolescents of single parent families, and particularly among those living with their father.

**Conclusions:**

Family structure inequalities in daily breakfast consumption increased between 1994 and 2010, while breakfast consumption across the population as a whole increased. As the proportion of young people living in an alternative family structure continues to grow it is important to understand why these inequalities have increased and how these may be overcome. Possible reasons for family structure inequalities and their increase in recent years are discussed.

## Background

Adolescence is a crucial time for developing and maintaining healthy eating habits, both in terms of the importance of essential nutrients during this period of growth and development, and because dietary habits formed during this life stage often track into adulthood [1]. Breakfast consumption is an important component of nutrition, and as part of a healthy diet and lifestyle, is thought to impact positively on children's health and well-being [2]. Skipping breakfast is associated with poorer nutritional habits, such as increased consumption of snacks and larger meal portions throughout the day [3,4], as well as increased risk of overweight [5,6]. By contrast, eating breakfast everyday is associated with having a healthy body weight [7] with evidence also suggesting improved cognitive function related to memory, test grades, and school attendance [2].

The role of family mealtime routines, such as breakfast, and their association with adolescent health has been studied previously [8,9]. In particular it has been shown that rituals and routines are important for the psychological health of all members of the family [10]. Young people therefore not only benefit directly, but also indirectly through the health and well-being of their parents. Family meals such as breakfast, therefore, not only improve the diet of young people [3], but also encourage positive relationships and communication between family members, reinforcing parental roles, a stronger family identity, connectedness and socialization of young people [9-11]. A daily ritual such as breakfast also helps to create a routine, whereby young people have structure in their day; for example, young people who eat breakfast are more likely to eat family meals and vice versa [12].

Evening family meals have previously been shown to be patterned by family structure in Scotland [13] and elsewhere [11], with children from both parent families more likely to eat a family meal every day. Breakfast may, in particular, be patterned by family structure, given that it is the meal most likely to be skipped and that irregular meal patterns are related to lifestyle factors [14]. Marital transition, associated with changes in the time spent on household chores and routines [15], may cause a disturbance to this family routine. A recent review of family correlates of breakfast consumption found living in a 2-parent family to be a predictor of adolescent breakfast consumption [16]. A further paper noted irregular breakfast consumption on weekdays among girls living in reconstructed families [17].

In recent years, many countries in Europe and the West have seen changes in family structure, with a rising proportion of young people living in alternative family set-ups to the traditional household composition of two biological parents [18]. In Scotland rates of marriage have increased since 1980, while rates of divorce have fluctuated [19]. Cohabitation has increased across Europe [18] and the proportion of children born and raised in cohabiting and reconstituted families in Scotland has increased [19]. Defining family structure as having one versus two parents ignores the complexity of increasingly diverse living situations, and for research purposes may be somewhat limiting. In particular, little is known about children of single father families, a growing subsample of the population.

Changes in family structure over recent decades are likely to have had an impact on adolescent breakfast consumption. Furthermore, alongside changes in family structure, the changing employment conditions in the UK over the same time period increasingly enable women to remain in employment while raising a family. This has resulted in a rise in female employment, with recent figures showing that 66% of women are in work and over 60% of 2-parent families have both parents in employment [18]. Juggling multiple roles and the resulting time constraints faced by working women are known to have an impact on family routines such as mealtimes, particularly in low- and moderate-income households [20,21]. In recognition of the importance of having breakfast, and the changing home and work environments, many schools now have breakfast clubs, before-school provision serving food to children who arrive early [22]. This concept originated in the US as a way of providing nutritional breakfasts to children from poorer areas, and has since been adopted in the UK, organised in accordance to individual schools' facilities and resources [23]. In 2010, 33% of primary and 58% of secondary schools in Scotland provided a breakfast club for pupils [24].

In light of what is already known about family structure inequalities in adolescent health, societal changes in family structure and the home environment, and the changing role of schools, the primary contributions of this study are to (1) describe the changes in breakfast consumption of adolescents in Scotland over a 16-year period, from 1994 to 2010, across 5 study points, (2) examine family structure inequalities in breakfast consumption, going beyond 'two parent families versus not', and (3) explore how the relationship between family structure and breakfast consumption has changed over time.

## Methods

### Data

This paper examines Scottish data from the 1994, 1998, 2002, 2006 and 2010 Health Behaviour in School-aged Children (HBSC) surveys. The research protocol was approved by University of Edinburgh ethics committee. At each survey, the population was stratified by education authority and school type, defined as state-funded or independent. Nationally representative samples of young people in school years: Primary 7, Secondary 2 and Secondary 4 were selected at each survey using systematic random sampling. Passive parental consent was used except where active consent was required. Questionnaires were completed anonymously in class under teacher supervision in school, between January and March so that the average ages of groups sampled were 11.5, 13.5, and 15.5 years.

### Outcome variable

Young people taking part in the HBSC survey were asked how often they ate breakfast through the week. In the 1994 and 1998 surveys this information was collected using the single question: 'During a normal week, how often do you usually have breakfast (with cereal, bread or cooked food)?' with response options *Every day/4 to 6 days a week/1 to 3 days a week/Hardly ever or Never*. This question changed in 2002 so that in the 2002, 2006 and 2010 surveys so that it consisted of two separate items relating to weekdays and weekends: 'How often do you usually have breakfast (more than a glass of milk or fruit juice)? with response options Weekdays: *I never have breakfast during weekdays/one day/two days/three days/four days/five days*. Weekend: *I never have breakfast during the weekend/I usually have breakfast on only one day of the weekend (Saturday OR Sunday)/I usually have breakfast on both weekend days (Saturday AND Sunday)*. These responses were summed to give a total range of breakfast consumed on 0-7 days per week. Breakfast consumption was then coded as a binary variable (Daily/Less than daily) for all survey years.

### Explanatory variables

Young people's age, sex and school grade were included in analysis. Age was centred, and a quadratic centred age term was included in analysis. Year of survey was also included.

Respondents were given a checklist of people from which they ticked those living in their main or only home. This included: mother, father, stepmother (or father's partner), stepfather (or mother's partner), siblings, grandparents, and adults other than their parents such as foster parents or care homes. Respondents were recorded as living with 'Both parents', a 'Single mother', 'Single father', in a 'Reconstituted family' or 'Other'.

### Statistical analysis

Preliminary analyses described the data, presenting frequencies for explanatory and outcomes variables. Proportions of young people within different family structures consuming breakfast on a daily basis at each of the five survey time points, weighted by age group and school type (state or independent), were calculated and presented. Logistic multilevel regression models were fitted for binary outcome variable daily breakfast consumption, using Markov chain Monte Carlo (MCMC) estimation methods in statistical package MLwiN 2.02 [25]. Estimates reported in the results are based on a chain of length of 50,000 following a burn-in of 5,000. Wald tests were carried out to identify significant parameter estimates and the Deviance Information Criterion (DIC) was used as a measure of model fit with a lower value of the DIC being favoured [26]. Models had three levels: Education Authority, school, and individual child. Fixed and random parameter estimates for models were tabulated, where fixed estimates were defined as the average effect across the entire population of Education Authorities, schools and individuals, while the random estimates described how these varied at each level. The models were first fitted, adjusting for age, sex, grade, year and family structure. Year was added first as a categorical and then as a continuous measure. Additionally, a marker variable for the change in item from 2002 onwards was added when continuous year was used. Interactions between age and year and sex and year were then added, as well as an interaction term between family structure and year to assess changes in the association between family structure and daily breakfast consumption. Identical logistic regressions were also carried out for two alternative breakfast consumption outcomes with cut-offs: breakfast eaten on 4 or more/less than 4 days per week (73%/27% of young people over the study period), and at least one day/no days per week (91%/9%).

## Results

### Daily breakfast consumption

The summary statistics presented in Table 1 were weighted by year to account for the fact that the sample size generally increased over time. On average across all years, 54% of young people consumed breakfast daily. However, when broken down by grade, this varied from 65% among P7 children to 45% among S4 pupils. Furthermore, 61% of boys ate breakfast daily compared with 48% of girls.

**Table 1:** Description of variables used in analysis

Measure	% ^a ^(N)
N	(26626)

Year:	

1994	17.5 (4664)

1998	20.3 (5410)

2002	16.2 (4314)

2006	21.8 (5808)

2010	24.1 (6430)

Sex:	

Boys	49.0 (13036)

Girls	51.0 (13590)

Grade:	

P7 (mean age 11.5)	35.1 (9335)

S2 (mean age 13.5)	33.1 (8819)

S4 (mean age 15.5)	31.8 (8473)

Family Structure:	

Both parents	70.6 (18795)

Single mother	16.0 (4266)

Single father	1.9 (509)

Step family	10.4 (2759)

Other	1.1 (297)

Breakfast consumption:	

Daily	54.3 (14453)

Less than daily	45.7 (12173)

Age (Mean^b ^(s.d.))	13.50 (1.67)

^a^Percentages for all but year variable were weighted by year

^b^Weighted by year

### Changes in daily breakfast consumption, 1994-2010

**Figure 1:**
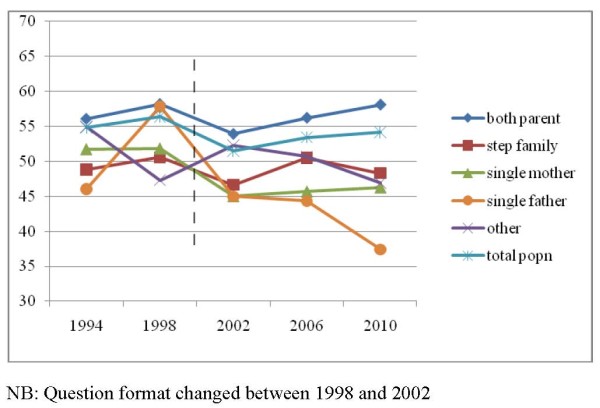
**Proportion of young people having breakfast every day by family structure, 1994-2010**.

Prevalence of daily breakfast consumption across Scotland increased between 1994 and 1998, reduced between 1998 and 2002, probably due to a change in the question wording in the 2002 survey, and increased again thereafter (Figure 1).

### Family structure

On average, across all time points, 71% reported living with both parents, while 18% reported living with one parent and 10% with a reconstituted family (Table 1).

### Changes in family structure, 1994-2010

**Figure 2:**
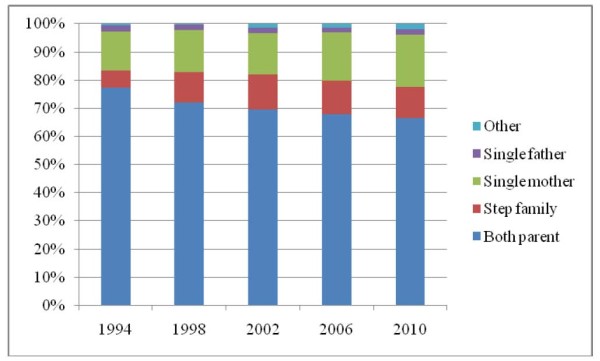
**Change in family structure in Scotland 1994-2010**.

Family structure, however, changed over time, so that in 1994, 77% lived with both parents, 6% lived in a reconstituted family and 14% with only their mother, compared with 67%, 11% and 19% respectively in 2010 (Figure 2). Although the proportions living with only their father and in alternative family set-ups not listed were small, these increased between 1994 and 2010, in particular proportion of 'other' tripled, from 0.6% to 1.9%.

### Daily breakfast consumption and family structure, 1994-2010

When split by family structure, daily breakfast consumption on average across this period was most prevalent among children of both parent families (Figure 1). Daily breakfast consumption followed a similar trend, albeit with lower proportions for children from reconstituted families, although with a reduction in prevalence between 2006 and 2010. Children from single mother families saw little change in prevalence from 2002 onwards, while children from single father families saw a reduction. Daily breakfast among children from 'other' family structure followed an opposite trend to that of the overall population, reducing between 1994 and 1998, increasing in 2002, and reducing thereafter.

### Results of regression analysis: Daily breakfast consumption and family structure

When the data were modelled, school grade became insignificant on addition of age which had a quadratic relationship with daily breakfast consumption (Model 1, Table 2). School grade was therefore removed from the model. The odds of girls eating breakfast daily were exp(-0.53) = 0.59 those of boys with a confidence interval (CI) of (0.56, 0.62). Odds of eating breakfast daily were also reduced for children from single mother (OR (CI) of 0.70 (0.65, 0.75)), single father (0.65 (0.54, 0.78)) and reconstituted families (0.78 (0.72, 0.85)) relative to those living with both parents.

**Table 2:** Multilevel logistic model for daily breakfast consumption outcomes, MCMC^a ^estimates (Monte Carlo standard error)

*Fixed effects*		Model 1^b^	Model 2^c^	Model 3^d^	Model 4^e^
Cons		0.38 (0.07)	0.46 (0.05)	0.53 (0.05)	0.49 (0.06)

Age		-0.21 (0.01)	-0.21 (0.01)	-0.10 (0.02)	-0.10 (0.02)

Age squared		0.04 (0.01)	0.04 (0.01)	0.04 (0.01)	0.04 (0.01)

Sex (ref: Male)	Female	-0.53 (0.03)	-0.53 (0.03)	-0.71 (0.05)	-0.71 (0.05)

Family structure (ref: Both parents)	Single mother	-0.35 (0.04)	-0.35 (0.04)	-0.35 (0.04)	-0.14 (0.07)

	Single father	-0.44 (0.09)	-0.44 (0.09)	-0.44 (0.09)	-0.13 (0.18)

	Step family	-0.25 (0.04)	-0.24 (0.04)	-0.24 (0.04)	-0.21 (0.09)

	Other	-0.20 (0.12)	-0.20 (0.12)	-0.21 (0.12)	0.04 (0.32)

Year (ref: 1994)	1998	0.22 (0.08)			

	2002	-0.03 (0.08)			

	2006	0.05 (0.08)			

	2010	0.10 (0.08)			

Year_cont_^f^			0.02 (0.01)	0.01 (0.01)	0.02 (0.01)

Age*Year_cont_^f^				-0.01 (0.001)	-0.01 (0.001)

Sex*Year_cont_^f ^(ref: Male*Year_cont_^f^)	Female*Year_cont_^f^			0.02 (0.004)	0.02 (0.005)

Family structure * Year_cont_^f ^interaction (ref: Both parents*Year_cont_^f^)	Single mother*Year_cont_^f^				-0.02 (0.01)

	Single father*Year_cont_^f^				-0.03 (0.02)

	Step family*Year_cont_^f^				-0.004 (0.01)

	Other*Year_cont_^f^				-0.02 (0.02)

*Random effects*					

Level 1 (child) variance^g^		1	1	1	1

Level 2 (school) variance		0.056 (0.011)	0.056 (0.011)	0.041 (0.010)	0.042 (0.010)

Level 3 (region)		0.028 (0.010)	0.026 (0.009)	0.026 (0.009)	0.026 (0.009)

$\bar{D}$^h^		34815.5	34818.2	34791.4	34776.9

*P_D_*^i^		255.2	254.7	214.2	219.9

*DIC*^j^		35070.7	35072.9	35005.6	34996.8

^a^via Markov chain Monte Carlo (MCMC); estimates are based on a chain of length of 50,000 following a burn-in of 5,000

^b^Model 1 adjusts for age, age^2^, sex, grade, family structure and year as a categorical variable

^c^Model 2 adjusts for age, age^2^, sex, grade, family structure, year as a continuous variable, and year marker following 2002 when the question on breakfast consumption changed

^d^Model 3 adjusts for age, age^2^, sex, grade, family structure, year as a continuous variable, year marker for years following 2002, and interaction terms between year and age and year and sex

^e^Model 4 adjusts for age, age^2^, sex, grade, family structure, year as a continuous variable, year marker for years following 2002, interaction terms between year and age and year and sex, and an interaction term between year and family structure. Interaction between family structure and year marker for years following 2002 is not significant

^f^Year_cont _refers to continuous Year variable, ranging between 1 (1994) and 17 (2010)

^g^Variance at the child level is constrained to 1

^h^$\bar{D}$ is the expectation of the deviance and is a measure of how well the model fits the data

^i^*P_D _*is the effective number of parameters

^j^*DIC *is the Deviance Information Criterion; the larger this is, the worse the model fit

### Results of regression analysis: Changes in daily breakfast consumption, 1994-2010

Overall, daily breakfast consumption in 2010 did not differ significantly from that of 1994 (Model 1, Table 2). However, breakfast consumption did increase significantly between 1994 and 1998 and when only 2002-2010 data were modelled, significant increases were seen. This suggests that the change in question format which occurred in 2002 may have caused people to answer the question differently. When year was modelled as a continuous variable (Model 2, Table 2) with a marker for 2002 data onwards, to account for the question format changed, a significant increase in breakfast consumption was observed, eg. between 1994 and 1995 odds increased by 1.02 (1.01, 1.03), by 2003 odds were 1.21 (1.09, 1.34) those of 1994, and by 2010, 1.37 (1.15, 1.64). A quadratic term for continuous year was not significant in the model.

### Results of regression analysis: Changes in the association between daily breakfast consumption, age and sex, 1994-2010

**Figure 3:**
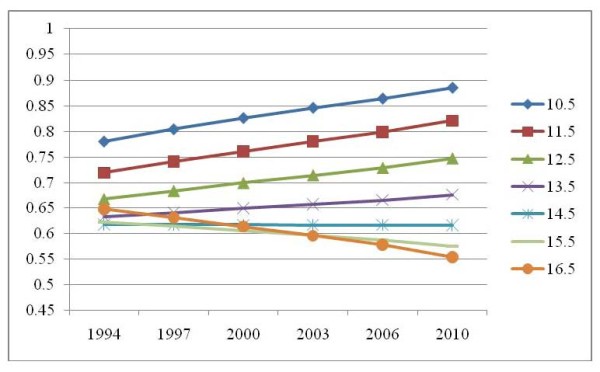
**Predicted probability of daily breakfast consumption for boys living in both parent families over time by age under Model 3, Table 2**.

Changes in daily breakfast consumption over time differed by age (Model 3, Table 2). Age is centred to avoid collinearity with its squared term, therefore the addition of the interaction with year results in a complex patterning of breakfast consumption over time. Predicted values under Model 3, Table 2 for boys of various ages living in both parent families are presented in Figure 3. Prevalence of breakfast consumption increased among adolescents under the age of 14.47 years over time, but decreased among those older than 14.47 years. Figures for boys from other family structures under this model would be identical but with a shift downwards on the y-axis. Interaction between year and age squared was not significant. Changes in daily breakfast consumption over time also differed by sex. While boys saw an increased odds of 1.01 in breakfast consumption between 1994 and 1995 (1.13 those of 1994 in 2003), girls saw increased odds of 1.03 between 1994 and 1995 (1.36 those of 1994 in 2003).

### Results of regression analysis: Changes in the association between daily breakfast consumption and family structure, 1994-2010

Adding an interaction term between family structure and year (Model 4, Table 2), showed a significant reduction in daily breakfast consumption among children of single parents when compared with children from both parent families. Under the model, between 1994 and 1995, the increase in breakfast consumption among children from single mother families was, on average, 0.98 (0.97, 0.99) and from single father families was 0.97 (0.94, 0.999) that of children from both parent families. By 2004 this difference widened, so that the increase for children from single mother families was on average, 0.79 (0.69, 0.90) that of children from both parent families, and the increase for children from single father families was 0.70 (0.49, 0.99) that of children from both parent families. By 2010 these differences widened further still. Random effects showed significant variance remained at school and region levels after adjustment for all explanatory variables in the logistic models (Table 2).

## Discussion

A recent review of child and adolescent breakfast consumption noted little research to date in the field of breakfast consumption and its association with family factors [16]. Only one of the included articles defined family structure in more detail than the number of parents in the home. However, families are complex and diverse. Differences in many child and adolescent health outcomes have been shown not only between one and two parent families but also between intact and reconstituted families [eg. [27-29]. Young people living in single father families report particularly poor mental health, low life satisfaction and disconnectedness [29-31] and it is therefore advised where possible to disaggregate lone parent families by parent gender. However, small proportions of lone father families often mean that lone parent families are grouped as one.

### Family structure inequalities in breakfast consumption

The current study was able to differentiate between both parent, reconstituted, single mother and single father families, grouping all other family types as 'other'. These included living with grandparents or other relatives, living in a care home and living with a mother and female partner. Analysis showed that family structure inequalities in breakfast consumption exist in Scotland with greater likelihood of eating breakfast daily among children living with both parents. The probability of eating breakfast daily was particularly low among children of single parent and reconstituted families, with the odds of daily breakfast lowest among those living with fathers only.

Explaining family structure inequalities is beyond the scope of this paper, however it is worth considering why breakfast consumption may be more easily achieved in some family types than others. One explanation for the difference in breakfast consumption by family type could be that family structure is a proxy for socioeconomic status. However, Pearson et al's systematic review showed that the relationship between breakfast consumption and SES was not consistent [16]. Moreover, the majority of studies included found an association with family structure even after adjustment for family SES. It could be that daily breakfast consumption is an indicator of a well-functioning ordered household, possibly one that is more likely to remain intact. Alternatively, the likelihood of eating breakfast daily may due to parenting style, also known to differ by family structure. For example, adolescents living in single parent families are thought to have more responsibilities, independence and decision-making power than those from two parent families [32]. As it is known that adolescents who make their own decisions about what they eat are 25% more likely to skip breakfast [12], this may explain the reduced probability of eating breakfast among single parent families. Furthermore, single father families have been characterised as 'uninvolved' compared with single mother and both parent families, and more 'permissive' and less 'authoritarian' compared with both parent families [30]. Pearson et al [33] found that adolescents who described their parents as authoritative ate breakfast on more days a week than those who did not.

A morning routine may be more easily established when there are two parents present. However, number of parents in the home does not in itself explain the difference, as the current study shows that children living in reconstituted families were significantly less likely to eat breakfast daily compared with both-parent families. This may be due to the fact that many live in more than one home, creating a more complex arrangement with potentially many step-siblings and where household functions may require renegotiation [34]. Pearson and colleagues [33,35] showed that number, as well as gender, of siblings in the home is associated with breakfast consumption. Furthermore, reconstituted households are more likely to have one or more strained parent-child relationships [29] also likely to impact on family meal frequency.

Lower levels of breakfast consumption among young people in single father families may be due to a lack of female influence on health behaviours. Previous research has shown men to rate health behaviours, such as food choice, as less important than women do [36]. Furthermore, women are more likely than men to report healthier food choices, explained in part by stronger health beliefs [37] and greater involvement in family meal preparation and buying of foods [38].

### Changes in breakfast consumption and family structure inequalities in breakfast consumption over time

Family structure inequalities in daily breakfast consumption increased between 1994 and 2010 with an approximately equivalent rise in daily breakfast consumption for young people living in both parent and reconstituted families, and a decrease in prevalence over time among those living in single parent families, particularly among those living only with their father. Increasing inequalities in breakfast consumption have occurred alongside a growing proportion of young people living in family structures other than the traditional 2-biological parent family. The population rise in breakfast consumption is therefore primarily due to the consistent increase in prevalence among those living with both parents.

On closer investigation it was found that the increase in breakfast consumption only occurred among those aged under 14.5 years, and more steeply among girls, probably due to a combination of factors. In particular, schools are likely to have played a large role in highlighting the importance of breakfast. The implementation of Health Promoting Schools in Scotland during the last two decades has brought the school environment to the forefront as a stage for health promotion [39]. As such, many schools in Scotland use breakfast clubs as a way of providing nutritious food to young people, as well as a medium for delivering the message of healthy eating behaviours [22,23]. Variation in adolescent breakfast consumption by school is evident in the current study. It is possible that implementation of breakfast clubs in schools has contributed to the increase in breakfast consumption over time, particularly among younger adolescents, both through provision of breakfast and promotion of its importance. Gender differences in health awareness discussed previously [36,37] may explain not only family structure differences between single mother and single father families, but also gender differences among adolescents in the increase of breakfast consumption over time.

In the current study, children of single parent families, and particularly single father families, saw a reduction in breakfast consumption prevalence over time and this warrants further investigation. Changing lifestyles may have influenced eating patterns differently in different family types. Recent figures have shown that the average time spent preparing food in Scotland has reduced from 60 min a day in 1980 to 18 min a day in 2007 [40]. In single parent households, where a larger number of responsibilities are placed on one parent, a routine such as breakfast may be less achievable. Lack of involvement among single fathers identified by Bronte-Tinkew et al. [30] may mean that public health strategies such as daily breakfast are not reaching single fathers or are not being undertaken, while families with stronger parental involvement adopt recommended health strategies. Alternatively, the decrease among children of single parent families may be due to the inception of breakfast clubs; Single parents may rely more on schools but children, particularly in secondary schools, may choose not to go when unsupervised.

### Recommendations and limitations

When asked about their main home and any second home they live in, it became clear that some young people had contact with more than just parent figures in the home. In some instances young people reported four parent figures, the maximum number allowed on the questionnaire. Perhaps even more relevant to the subject of a family routine such as breakfast, were the number of young people responding that they lived in a second home. Future research is required to tease out the underlying factors, such as number of homes and number of parent figures as well as parent-child relationships, which may influence daily breakfast consumption within differing family types. It is also recommended that future research investigates the impact of school level initiatives such as breakfast clubs on adolescent breakfast consumption and family structure inequalities in breakfast consumption.

There are some limitations in the definition of breakfast consumption. The change in question format between 1998 and 2002 may have affected the trend in proportions presented over time. However this was adjusted for in the regression analysis. Ideally, raw frequency of breakfast consumption would have been modelled. Alternatively, it may have been preferential to limit the study to weekday breakfast consumption as this has been the focus in recent publications [16]. Using a binary outcome, daily breakfast consumption versus breakfast consumption on a fewer number of days, groups together those who ate breakfast on 5 or 6 days a week with those who never ate breakfast at all. However, the categorical format of the survey question in 1994 and 1998 meant that modelling of raw (or weekday only) data could not be carried out. The data were however re-modelled for outcome variables breakfast on 4 or more/less than 4 days per week and at least once a week/never (tables are available from the authors on request), and although the odds changed a little, the results and conclusions remained the same; for all three outcomes the final model showed significantly poorer results over time for those from single father and single mother families relative to those living with both parents.

## Conclusions

Family structure inequalities in daily breakfast consumption increased between 1994 and 2010, while breakfast consumption across the population as a whole increased. As the proportion of young people living in alternative family structure continues to grow it is important to continue monitoring changes in breakfast consumption by family type, alongside overall population trends. Further work is recommended to investigate familial factors associated with breakfast consumption which may explain the family structure differences observed. School breakfast clubs and related emerging health promoting activities must also be considered, as these may also be relevant in understanding why these inequalities have increased and how these may be overcome.

## Competing interests

The authors declare that they have no competing interests.

## Authors' contributions

KAL was responsible for conception of the study and statistical analysis. KAL and JK were involved in drafting the manuscript. CC was involved in revising the manuscript critically and producing the final version. All authors read and approved the final manuscript

## Pre-publication history

The pre-publication history for this paper can be accessed here:

http://www.biomedcentral.com/1471-2458/12/228/prepub
